# Recent advances in the management of migraine

**DOI:** 10.12688/f1000research.9764.1

**Published:** 2016-11-21

**Authors:** Mark Obermann, Dagny Holle

**Affiliations:** 1 Center for Neurology, Asklepios Hospitals Schildautal, Seesen, Germany; 2 Department of Neurology and West-German Headache Center, University of Duisburg-Essen, Essen, Germany

**Keywords:** migraine management, recent advances, CGRP, lasmiditan, vagus nerve stimulation

## Abstract

Migraine remains one of the most disabling disorders worldwide. The high prevalence in the general population and the often-delicate treatment of patients account for that. Therapeutic management of migraine relies mainly on non-specific medical treatment and is affected by low patient adherence to the treatment regimens applied. The introduction of specific anti-migraine treatment occurred over 20 years ago when the first triptan was approved by regulatory authorities (sumatriptan, 28 December 1992). Triptan use is limited by side effects, time- and frequency-restricted application, and the risk of developing medication overuse headache. Within the past few years, new and promising drugs such as more specific 5-HT 1F receptor agonists (that is, lasmiditan) and monoclonal calcitonin gene-related peptide (CGRP) receptor antibodies entered advanced development phases while non-invasive neuromodulatory approaches were suggested to be potentially effective as non-pharmaceutical interventions for migraine.

## Introduction

The Global Burden of Disease Survey 2010 ranked migraine the second most common disease worldwide ^1^, with a prevalence of 14.7%. Migraine is associated with a substantially reduced quality of life for the individual and a high level of economic burden for society. Migraine was recognized among the seven highest specific causes of disability worldwide. The costs of migraine in the European Union were estimated to exceed 100 billion euros per year ^2^. Although most of these costs are related to economic deficits such as inability to work and absence from work, one major factor is medical treatment. Within the past decade, different substances were introduced to treat migraine but with limited success in some patients. Only triptans have been developed for the treatment of migraine specifically and although their efficacy is generally good in most patients, their usage is limited by side effects and the number of times they can be used safely before exposing the patient to the risk of cardiac side effects or the development of medication overuse headache. After several setbacks in the development of novel promising drug classes that led to the discontinuation of the respective development program (that is, the calcitonin gene-related peptide [CGRP] receptor antagonists olcegepant and telcagepant and the glutamate receptor antagonist BGG492), there are now several quite promising therapeutic agents emerging from phase II and early phase III studies that may well fulfill these aims.

Even though final confirmation of efficacy for most of the drugs discussed below will have to follow within the next few years, they will likely change the way we treat migraine in the future and considerably add therapeutic power to the specific treatment of migraine.

Non-pharmaceutical interventions such as behavioral therapy and non-invasive neuromodulatory approaches such as vagus nerve stimulation (VNS) and transcutaneous supraorbital nerve stimulation were successfully tested to treat migraine and represent a treatment alternative for those patients unwilling or unable to manage their disorder pharmacologically.

## 5-hydroxytriptamine 1F receptor agonists

Lasmiditan is a 5-hydroxytriptamine ****(HT) 1F receptor agonist that works similarly to triptans (5-HT 1B/1D agonists) but without the vasoconstrictive side effects well known to this substance class ^3^. It was given the generic stem name “ditan” to distinguish it from the “triptans” and other drug classes. The results of the SAMURAI (A **S**tudy of two doses of l **A**s **M**iditan compared to placebo in the ac **U**te treatment of mig **RAI**ne) trial were presented recently ^4^. The SAMURAI trial was a randomized, double-blinded, placebo-controlled parallel group phase III clinical trial that compared 100 and 200 mg oral administration of lasmiditan with placebo for 2-hour pain freedom from migraine headache as the primary endpoint. The trial randomly assigned 2,231 migraine patients in the United States, including those patients with cardiovascular risk factors that would not be appropriate for treatment with triptans. The mean age of randomly assigned patients was 41.6 years, 83% were women, and the mean disease duration was 19 years. They had an average of five migraine attacks per month and an average MIDAS (migraine disability assessment) score of 31. One-quarter of patients used prophylactic migraine medication, and 82% of patients had cardiovascular risk factors. The most common ones were obesity, family history of coronary artery disease, smoking, hypertension, hyperlipidemia, and diabetes mellitus type 2. Patients also had cardiovascular conditions such as arrhythmias, mitral valve disease, angina, atrial fibrillation, congestive heart failure, prior myocardial infarction, or ischemic stroke. A single migraine attack was treated and 28.2% (odds ratio 2.2, 95% confidence interval [CI] 1.6 to 3.0, *P* <0.0001) of patients were pain-free at 2 hours with 100 mg lasmiditan, 32.2% (odds ratio 2.6, 95% CI 2.0 to 3.6, *P* <0.001) were pain-free with 200 mg, and only 15.3% with placebo, thus reaching the primary endpoint. These results are in a comparable efficacy range of key clinical trials done with triptans without having access to direct comparison trials. Lasmiditan was generally well tolerated with mild or moderate side effects. The most common side effects were dizziness, paresthesia, somnolence, nausea, fatigue, and lethargy ( Table 1). In conclusion, lasmiditan showed a good treatment effect in highly affected migraine patients that is comparable to triptans but with a much better cardiovascular side effect profile. It may become a valuable treatment option for patients at risk for cardiovascular events due to vasoconstriction.

**Table 1.** Most common side effects of lasmiditan in the SAMURAI trial ^4^.

Side effect	100 mg, n = 630	200 mg, n = 609	Placebo, n = 617
Dizziness	75 (11.9%)	94 (15.4%)	19 (3.1%)
Paresthesia	36 (5.7%)	46 (7.6%)	13 (2.1%)
Somnolence	33 (5.2%)	32 (5.3%)	14 (2.3%)
Nausea	16 (2.5%)	29 (4.8%)	9 (1.5%)
Fatigue	24 (3.8%)	18 (3.0%)	1 (0.2%)
Lethargy	12 (1.9%)	14 (2.3%)	1 (0.2%)

These promising results will have to be confirmed by the ongoing SPARTAN (Three Doses of Lasmiditan [50, 100, and 200 mg] Compared to Placebo in the Acute Treatment of Migraine) trial that uses the same endpoints and same statistical power. It also tests the 50 mg dose of lasmiditan in order to establish the lowest effective dose for an even better adverse event profile ^4^.

## Monoclonal calcitonin gene-related peptide antibodies

Four monoclonal antibodies targeting the CGRP pathways were introduced within the past few years and all have successfully completed their phase II study and are entering phase III clinical trials at the moment ( Table 2; ^5– 10^). The main difference between the different substances is the frequency and route of administration (subcutaneously or intravenously). AMG 334 targets the CGRP receptor, whereas the other three antibodies bind the CGRP peptide. The CGRP receptor is a complex transmembrane receptor (CLR/RAMP 1 complex) that is 5,000 times more selective for CGRP than other related receptors in this class. The 70 mg dose of AMG 334 was effective with a mean monthly migraine day reduction of 3.4 days compared with placebo (−2.28 days; *P* = 0.021), whereas the lower doses with 7 and 21 mg, respectively, were not significant ^6^. Patients received subcutaneous injections every 4 weeks. The double-blind treatment phase was completed by 448 (93%) patients. Adverse events were mild to moderate in severity and did not differ between the active drug and placebo. The most common complaints were nasopharyngitis and fatigue. Importantly, given the experience with small-molecule antagonists, no hepatotoxicity was observed in these patients ^6^.

**Table 2.** Monoclonal antibodies targeting the calcitonin gene-related peptide pathway.

**LY2951742**	Superior to placebo (number of migraine headache days −4.2 days after 12 weeks compared with baseline, placebo −3.0 days; *P* = 0.003) ^8^
**ALD403**	Superior to placebo (comparing baseline migraine days to weeks 5–8 reduction of 5.6 migraine days compared with 4.6 days in the placebo group; *P* = 0.0306) ^7^
**AMG 334**	Superior to placebo (mean monthly migraine days −3.4 days compared with placebo; −2.28 days; *P* = 0.021) ^6^
**TEV-48125 (LBR-101)**	Superior to placebo (high-frequent episodic migraine reduction in headache days of −4.86 days for the 225 mg group and −4.80 days for the 600 mg group; both *P* <0.0001) ^9^
**TEV-48125 (LBR-101)**	Superior to placebo (chronic migraine −6.04 days in the 675 mg group, *P* = 0.0386, −6.16 days in the 900 mg group, *P* = 0.0057) ^10^

ALD403 is one of the antibodies binding CGRP ^7^. One gram was administered intravenously every 4 weeks and led to a reduction of 5.6 migraine days compared with 4.6 days in the placebo group ( *P* = 0.0306). Eleven percent of 67 treated patients showed a 100% response (that is, no more migraine attacks) after 12 weeks ^7^. Similar efficacy was observed with the subcutaneous administration of 150 mg LY2951742 every 2 weeks compared with placebo ^8^. The number of migraine headache days was decreased by 4.2 days after 12 weeks compared with baseline, whereas placebo reduced the headache days by only 3.0 days ( *P* = 0.003). The 100% responder rate was 15% in a *post hoc* analysis, and no serious adverse events occurred on treatment ^8^.

TEV-48125 was tested in high-frequency episodic migraine as well as chronic migraine in two separate controlled clinical trials ^9, 10^. Episodic migraine patients with 8 to 14 headache days per month received either 225 or 600 mg TEV-48125 every 4 weeks subcutaneously and showed reductions in headache days of 4.86 days in the 225 mg group and 4.80 days in the 600 mg group, whereas placebo reduced the headache days by 3.10 days (both *P* <0.0001). The most common treatment-related adverse events were mild injection site pain or erythema without differences between the groups ^9^.

TEV-48125 was equally effective in chronic migraine patients who had more than 15 headache days per month. The number of headache days after 12 weeks was reduced by 6.04 days in the group receiving up to 675 mg ( *P* = 0.0386), 6.2 days in the 900 mg group ( *P* = 0.0057), and only 4.2 days in the placebo group. Four patients reported serious adverse events, one in the placebo group. One patient had acute kidney stone pain, one patient developed pneumonia after influenza infection, one had irritable bowel syndrome, and one had an episode of depression with suicidal ideation. None of these events was related to the study drug by the investigators ^10^.

## Non-invasive neuromodulatory approaches: transcutaneous vagus nerve stimulation

The EVENT (Chronic migraine headache prevention with noninvasive VNS) study investigated the efficacy and safety of non-invasive, transcutaneous VNS for the treatment of chronic migraine ^11^. The stimulation device delivers a low voltage (peak of 24 V) and maximum output current of 60 mA to the right side of the neck close to the vagus nerve. Three 2-minute treatment sessions were applied per day over 2 months followed by a 6-month open-label extension phase. In total, 59 patients (30 VNS and 29 sham) were evaluated in an intention-to-treat analysis. Although the mean change in headache days from baseline after 2 months was not different between groups ( *P* = 0.56), the number of headache days continued to decrease in the open-label phase with a mean of 17.2 (95% CI 13.8 to 20.5) headache days and a mean change from baseline of −3.6 (95% CI −6.3 to −0.87; *P* = 0.02) after 8 months of treatment. Adverse events that were deemed possibly related to the device included eye twitch, facial pain/numbness, paresthesia, treatment site skin reaction, worsening migraine, and gastrointestinal symptoms. All adverse events were distributed equally across groups. Adherence was over 90% in both groups throughout the study. Transcutaneous VNS may become an interesting adjunct non-pharmacological treatment option for the treatment of chronic migraine. Further studies with more patients and a longer blinded study period will have to determine the value of this treatment ^11^.

## Conclusions

The next couple of years will be an exciting time for patients with migraine and their physicians. The range of different treatment modalities considerably widened within the past few years and may continue to do so in the years to come. Medications with new mechanisms of action and fewer side effects may prove to be safe and effective for the treatment of episodic and chronic migraine. Additionally, non-invasive neuromodulation may prove to be a genuine therapeutic asset in the management of migraine.
